# Modular and Self-Contained Microfluidic Analytical Platforms Enabled by Magnetorheological Elastomer Microactuators

**DOI:** 10.3390/mi12060604

**Published:** 2021-05-23

**Authors:** Yuxin Zhang, Tim Cole, Guolin Yun, Yuxing Li, Qianbin Zhao, Hongda Lu, Jiahao Zheng, Weihua Li, Shi-Yang Tang

**Affiliations:** 1 Department of Electronic, Electrical and Systems Engineering, University of Birmingham, Edgbaston, Birmingham B15 2TT, UK; YXZ048@student.bham.ac.uk (Y.Z.); TXC991@student.bham.ac.uk (T.C.); JXZ057@student.bham.ac.uk (J.Z.); 2 School of Mechanical, Materials, Mechatronic and Biomedical Engineering, University of Wollongong, Wollongong, NSW 2522, Australia; gy417@uowmail.edu.au (G.Y.); yl452@uowmail.edu.au (Y.L.); hl108@uowmail.edu.au (H.L.); 3 School of Electrical and Electronic Engineering, Nanyang Technological University, Singapore 639798, Singapore; qianbin.zhao@ntu.edu.sg

**Keywords:** microfluidics, self-contained system, lab-on-a-chip, magnetorheological elastomer, actuators

## Abstract

Portability and low-cost analytic ability are desirable for point-of-care (POC) diagnostics; however, current POC testing platforms often require time-consuming multiple microfabrication steps and rely on bulky and costly equipment. This hinders the capability of microfluidics to prove its power outside of laboratories and narrows the range of applications. This paper details a self-contained microfluidic device, which does not require any external connection or tubing to deliver insert-and-use image-based analysis. Without any microfabrication, magnetorheological elastomer (MRE) microactuators including pumps, mixers and valves are integrated into one modular microfluidic chip based on novel manipulation principles. By inserting the chip into the driving and controlling platform, the system demonstrates sample preparation and sequential pumping processes. Furthermore, due to the straightforward fabrication process, chips can be rapidly reconfigured at a low cost, which validates the robustness and versatility of an MRE-enabled microfluidic platform as an option for developing an integrated lab-on-a-chip system.

## 1. Introduction

Microfluidics is a systematic science and technology that is able to accurately manipulate and control small-scale amounts of fluids and particles at micron and submicron dimensions [1]. Microfluidics has many advantages, including (1) the capability to lower the usage of samples and expensive reagents and to separate and detect samples with high sensitivity and resolution; (2) short time for processing and analysing; (3) integrated reference systems with little human involvement, decreasing odds of sample contamination; and (4) small footprints for the analytical platforms, offering potential for point-of-care (POC) applications [2,3,4]. However, in order to accomplish liquid manipulation in small scale, bulky and expensive instruments such as syringe pumps and centrifuges are necessary, which prevent unleashing the unparalleled potential of microfluidic systems. To overcome this restriction, numerous attempts have been made to develop a self-contained microfluidic system, which integrates functional microactuators and control module into a miniaturised system for performing a total analytical process on a microfluidic chip [5,6,7,8]. The concept of a self-contained microfluidic system significantly accelerates the advance of microfluidic-based POC systems and expands its utilisation outside research laboratories.

Current self-contained microfluidic systems are usually designed for very specific processes, where all manipulators, detection sensors and reactors are specialised for a single application [6]. The format of monolithic chips makes the fabrication process complicated and narrows the available range of applications. Once the platform is constructed, it is expensive and time-consuming to reconfigure or add additional functions. Recent development of rapid prototyping as an alternative method for creating microfluidic devices provides a cost-effective solution to simplify multistep microfabrication in clean room facilities [9,10,11,12]. These new approaches utilise commercially accessible materials and low-cost fabrication equipment outside of the cleanroom, such as laser cutters, plotter cutters and 3D printers, which provides opportunities to quickly reconfigure a self-contained platform at low cost with modularised components. Numerous novel microchannel fabrication methods have been developed, including sandwiched adhesive tapes between two pieces of plastic with access ports to serve as liquid reservoirs [13,14,15], 3D-printed resin chips [16,17,18] and micropumps and micromixers working with magnetorheological elastomers (MRE) [19] .

MRE can be defined as follows: a composite material which is composed of a non-magnetic polymeric matrix and micro-sized ferromagnetic particles [20]. The presence of a controllable magnetic field can modify its mechanical properties. Its unique advantages, including low cost, inexpensive processing, readily tuneable mechanical properties and diverse configurability, offer potential for use in various disciplines, such as automobile engineering and civil engineering [20,21]. By combining the MRE units with microfluidic chips, the fluids flowing in the microchannels can be manipulated by changing magnetic fields. As a result, MRE has been used in microfluidic systems to provide an actuation mechanism for controlling fluid [19], manipulating particles and droplets [22,23] and actuating interconnects [24]. Among the emerging MRE-enabled microfluidic devices, Tang et al. discovered a novel liquid manipulation mechanism using MRE actuators and designed micropumps and a micromixer [19]. Such actuators demonstrate its versatility, providing an alternative to conventional microfluidic pumps and mixers. In addition, by integrating microactuators, a modularised platform can be rapidly built and reconfigured for other purposes. Although this MRE-enabled platform is simple and robust, the requirement of a soft lithography process limits its application in low-resource sites. 

To overcome the drawbacks of conventional platforms, here, three essential components for liquid manipulation in microfluidic chips, including a pump, mixer and valve, were created using MRE, and they were integrated into poly (methyl methacrylate) (PMMA)-based microfluidic chips. A simple and rapid laser-cutting-enabled fabrication technique was provided without the need of any microfabrication process. An integrated control platform was established, containing a MRE driving system, a microcontroller and a precise valve positioning system. By combining microfluidic chips and the control platform, an insert-and-use modular system was built, which demonstrates the feasibility of the platform for self-contained microfluidic applications.

## 2. Materials and Methods

Carbonyl iron microparticles (2–5 µm) and polydimethylsiloxane (PDMS) kit were purchased from Sigma-Aldrich, Sydney, Australia. A pulse width modulation (PWM) speed controller (12 V, 8 A), Arduino microcontroller board (Uno, V3), H-bridge motor controlling shield and RGB Colour Sensor (TCS34725, Adafruit, New York, NY, USA), DC motors (12 V, 50 kg/cm at 55 RPM), digital tachometer and permanent magnets were purchased from Jaycar Electronics, Sydney, Australia. The Digital Microscope, double-sided adhesive (300 LSE, 3M Ltd., St. Paul, MN, USA) and PMMA board were purchased online. The design of the microchannel and PMMA frames were first drawn in AutoCAD. The vector drawings were cut using a CO_2_ laser engraver and cutter system (Versa Laser System, Model VLS 3.50, Universal Laser System, Ltd., Scottsdale, AZ, USA). The pre-set parameters were used for cutting the PMMA board in different thicknesses. For double-sided adhesive, the laser power was optimised and finally set to ~5 W to effectively cut the adhesive sheet across with smooth edges. The lines in the drawing defined the cutting path of the laser beam; therefore, the verification and modification of different channel designs are simple and straightforward. The MRE was made by mixing polydimethylsiloxane (PDMS) (base/curing agent ratio = 20:1) with carbonyl iron microparticles (diameter of 2–5 µm) with a weight ratio of 1:4. The obtained mixture was poured and cured onto a PMMA mould at 70 °C for 6 h to fabricate the microactuators. A schematic of MRE production can be found in Figure S1 in Supplementary Materials. Syringe pumps (Legato 100, KD Scientific, Holliston, MA, USA) were used to inject the liquids into the chip for experiments examining the mixing performance. The rotating speed of the magnet holder was measured using a tachometer. The image processing toolbox of MATLAB 2021a software package (MathWorks, Inc., Natick, MA, USA) was used to obtain the profile of flows inside the microchannels.

## 3. Results and Discussion

**Figure 1:**
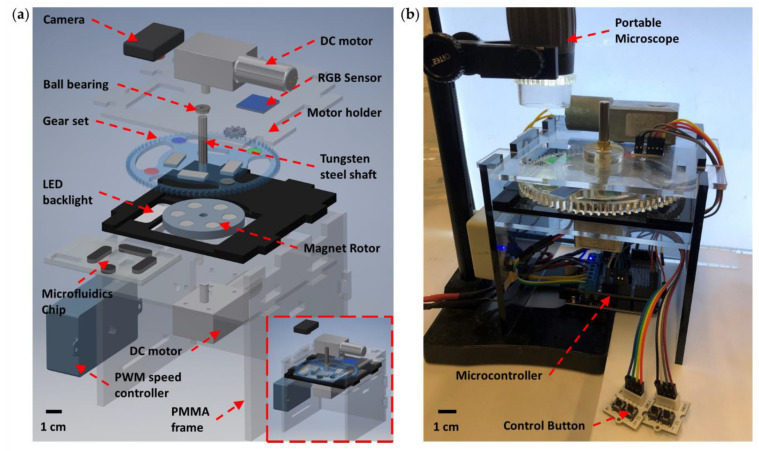
Schematic illustration of the self-contained microfluidics platform enabled by magnetorheological elastomer (MRE). (**a**) Exploded schematic representation of the integrated platform. The lower inset shows the assembled model of the system. (**b**) Actual image of the assembled system integrated with a portable digital microscope.

The MRE micropump was able to be actuated by a number of rotating magnets, as shown in Figure 1a and Video S1. Six cylindrical neodymium magnets (10 mm diameter and 7 mm thickness) were placed in the holes on a polymethylmethacrylate (PMMA) circular holder (50 mm diameter and 6 mm thickness). A low-carbon steel sheet (15 mm diameter and 2 mm thickness) was attached to the bottom of the holes to hold the magnets. The measured magnetic field intensity on the top of a magnet was ~350 mT. The magnet holder was rotated by a DC motor (12 V input voltage) equipped with a reduction gearbox. The speed of rotation was controlled using a PWM controller. In addition to this, a LED backlight and a portable microscope or camera comprise the analysis and detection module. An 8:1 transmission gear set and a DC motor on the top of the chip establish the controllable valve system, covered in detail in the following section. An Arduino microcontroller attached with a H-bridge shield for motor controlling, a RGB colour sensor for positioning and four switch buttons for manual input make up the control module. The entire system was assembled on a PMMA frame to establish a miniaturised and integrated platform (Figure 1a inset). The platform has a square base, with sides of 13.5 cm length and a height of 16.5 cm. The total weight of the platform is less than 1 kg. The controller platform could be further reduced in size by replacing driving components and optimising inner structures in a future study, which would further improve its portability. The actual image of the completed self-contained system is shown in Figure 1b, in which MRE microactuators such as pumps and mixers can be placed in a modular chip and actuated on the top of the system; most importantly, the microfluidic chips can be readily integrated with this platform, enabling a versatile modular platform for a wide range of applications. A detailed investigation into the MRE microactuators and integrated platform is provided in the following sections.

### 3.1. Micropump

**Figure 2:**
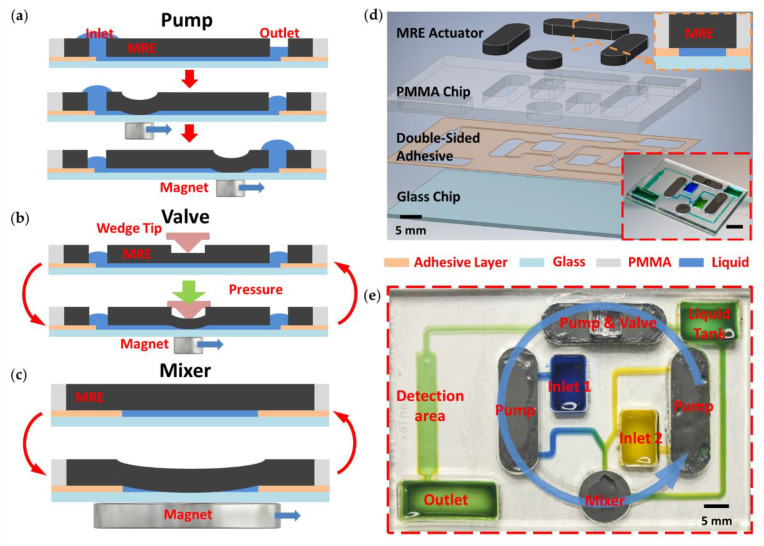
Schematic illustration of the microfluidic chip design and the working principle of MRE actuators. Schematic of the working mechanism of a MRE (**a**) micropump, (**b**) microvalve and (**c**) micromixer. (**d**) Exploded schematic representation of one design of the MRE microfluidic chip. The upper insert illustrates the cross-sectional view for a MRE actuator. The lower inset shows actual image of an assembled chip (Scale bar = 10 mm). (**e**) Actual image of the assembled chip integrated with three MRE pumps, one mixer and one valve.

In previous work, MRE-based microactuators including a pump and mixer for microfluidic applications were developed [19]. The cross-sectional schematic shown in Figure 2a depicts the working mechanism of a MRE micropump. The pump chamber is deformed upon exposure to a nonuniform external magnetic field provided by a permanent magnet. This induces a temporary collapse of part of the chamber. The collapsed MRE is able to ‘push’ liquid from the inlet towards the outlet as the magnet moves along the chamber, causing pumping similar to a peristaltic pump. This process was repeated continuously in order to achieve a continuous pumping of liquid. 

### 3.2. Microvalve

Figure 2b shows the working principle of the MRE microvalve. Based on the principle of a MRE micropump, an external pressure can be applied on the MRE to seal the chamber and stop the liquid flow. The mould used had a groove in the centre. A wedge was used and inserted in the groove to apply pressure to the MRE. The groove in the MRE meant it had a thinner layer, which required a smaller force to collapse the chamber. In addition, the chamber also has a reduced width in the middle to provide a better sealing performance. When the valve is activated, the wedge pushes down until the MRE collapses and the chamber is sealed. 

### 3.3. Micromixer

Figure 2c depicts the working mechanism of the MRE micromixer. The presence of a magnet under the glass substrate is able to generate a force to induce the deformation of the chamber. Oscillation of the MRE can be generated with the periodical presence and absence of magnets underneath, inducing chaotic advections within the chamber and triggering a mixing effect between the incoming flows.

### 3.4. Integrated Device

As part of the self-contained microfluidic platform, a chip integrated with MRE microactuators is shown in Figure 2d. There are four key elements on the microfluidic chip; these are: a 2 × 3-inch glass chip for the substrate, a laser-cut double-sided adhesive for the microchannels, a PMMA chip for the liquid reservoirs and sealing channels and MRE actuators for manipulating the flow. All components are bonded together through plasma treating with double-sided adhesive. In addition to the simple fabrication of the MRE actuators, the PMMA chip and the adhesive layer attached on it were all fabricated using a computer-controlled CO_2_ laser cutting machine. The MRE actuators were cured in a PMMA mould and then inserted into the chambers on the PMMA chip. The cured MRE surface demonstrated a relatively smooth surface at the bottom, where the microparticles were sparsely distributed and fully encapsulated by PDMS. The well-encapsulated iron particles under the PDMS surface allows the post-bonding process and prevents contamination [19]. The MRE has an oxygen plasma treated bottom surface, which, in combination with the double-sided adhesive, gives high bond strength, ensuring its long-term operation and good chemical and humidity resistance. The soft elastomers also assist in sealing the chamber to prevent leaking. A step-by-step fabrication process is given in Figure S2 in Supplementary Materials. Figure 2e illustrates a typical microfluidic chip design integrating the MRE micropump, micromixer and valve. 

The formation of bubbles can seriously block the microchannels and affect the accuracy of analysis. Therefore, in order to minimise bubble formation, the integrated chips were initially loaded with a small amount of buffer liquid in on-chip reservoirs and placed into a vacuum chamber for 5 min. Due to the change in pressure, the buffer liquid flowed into MRE actuator chambers and the bubble can be removed. 

In order to use the platform, a few steps are required. First, prepared liquid samples are loaded into two inlet reservoirs. The micropumps are then simultaneously driven by the spinning magnet rotor beneath (see Figure 2e, the blue arrow indicates the rotation direction). Along with the fluids being pumped, the MRE micromixer is activated and continually mixes the two incoming liquids with high mixing efficiency. The MRE valve downstream is activated (closed) at the beginning, which allows for storage of a well-mixed sample in the liquid tank. When the liquid tank has stored sufficient samples, users are able to manually or automatically release the valve so that the pump can start working and fluid will go through the detection area for further evaluation and examination. The working flow was demonstrated with blue and yellow food dyes (see Video S2).

The cost of a disposable integrated chip was estimated to be GBP 2.77. A detailed cost estimation list is presented in Table S1 in the Supplementary Information. The cost of the control platform is calculated to be GBP 55, including main components such as DC motors with gear reduction boxes (GBP 20) and the Arduino microcontroller (GBP 17). The material cost can be further optimised with a more cost-effective substrate.

**Figure 3:**
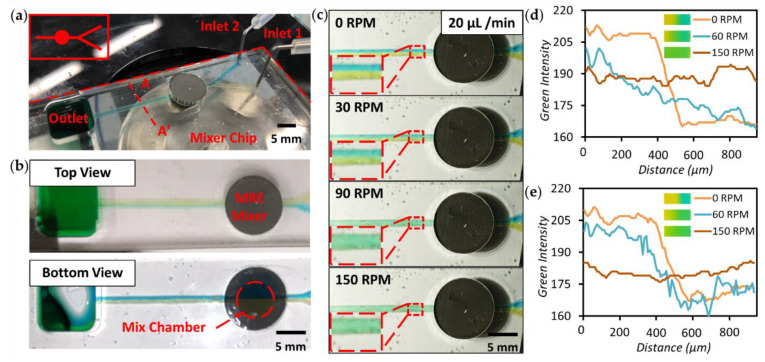
Experiments for examining on-chip MRE micromixer performance. (**a**) Actual image for the integrated MRE micromixer. The upper insert shows the microchannel design. (**b**) Top and bottom views of the micromixer channels. (**c**) Snapshots showing the mixing performance at inlet flow rates of 20 µL/min. Plot of the green colour intensity profiles along A-A′ shown in (**a**) across the microchannel at a flow rate of (**d**) 40 µL/min and (**e**) 100 µL/min with the magnet rotor rotating at speeds of 0, 60 and 150 RPM. Images of the channels at different RPMs are shown next to the legend.

To study the performance of the MRE mixer, we designed a simple chip with a mixer for mixing two incoming flows, as shown in Figure 3a. The upper insert shows the schematic of the channel design. The two inlets of the mixer were connected to syringe pumps to control the flow rate; deionised (DI) water mixed with yellow and blue food dyes was injected into the mixing chamber. In Figure 3b, the top and bottom view of the micromixer are shown before mixing, where the laminar flows of the two different colour liquids can be observed. The red dashed circle shows the location of the 6 mm diameter circular mixing chamber. When a flow rate from 20 to 120 µL/min was applied to each inlet before driving the mixer, the flow was purely laminar due to its low Reynolds number. The merging of the two colours can only be observed at the boundary between incoming flows, as evidenced by the formation of a clear boundary between the yellow and blue coloured solutions along the middle of the microchannel (see Figure 3c–e and their insets). When the magnet rotor rotates at 30 RPM, the micromixer is activated and the colour change becomes more gradual. When the rotor speed is further increased, a uniformly distributed colour gradient is formed. A homogeneous distribution of green colour flow can be obtained at higher rotor speeds. Compared to the MRE micromixer in previous work [19], the mixing efficiency is significantly improved at a high flow rate of 40 and 100 µL/min (Figure 3d,e). This is most likely due to the larger size of the MRE actuator and the optimised stiffness of the MRE. The mixing performance analysis is shown in Figure 3d,e. A MATLAB image process toolbox was used to calculate the intensity of red, green and blue along the line A-A′ (see in Figure 3a), which is located across the microchannel at 4 mm downstream of the micromixer. A green density profile is shown for different rotating speeds of the magnet rotor at a flow rate of 40 and 100 µL/min. A sudden change between two colours is shown in the profile at 0 RPM, which indicates laminar flow in the microchannel. The colour intensity profiles gradually become uniform with higher rotor speeds, demonstrating the mixing capability of the MRE micromixer in an integrated microfluidic chip. When a flow rate of 100 µL/min was applied, the mixing efficiency slightly decreased due to the reduced time of liquid staying in the mixing chamber. For applications with higher flow rates and mixing performance requirements, modifications such as increased mixer size or multiple micromixers could be utilised. The MRE micromixer can be used as a powerful pre-process module for sample preparation and integrated with other systems.

**Figure 4:**
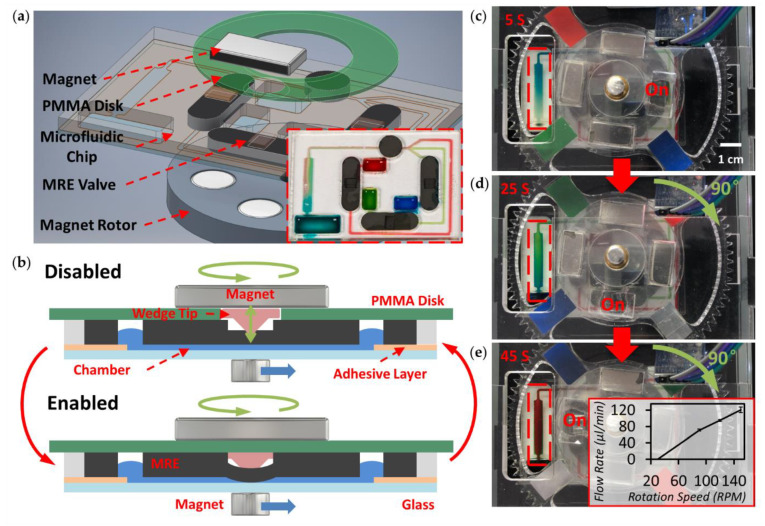
Schematic of setup for integrated MRE valve. (**a**) Exploded schematic representation of the valve setup. The lower inset shows the actual image of the chip design for sequential pumping. (**b**) The disabled and enabled working states of the MRE valve. (**c**–**e**) Sequential snapchats showing achievement of sequential pumping. The lower insert plot shows the flow rate vs. rotation speed of the magnet rotor.

In order to control the valving system, a gear set (transmission ratio of 8:1) with magnets to supply external pressure, a DC motor as the driver and a RGB colour sensor for position feedback were included in the system. The attractive force between the upper rectangular magnet and the lower magnet rotor provides external pressure. The rectangular magnets were inserted and fixed into the PMMA gear set, ensuring precise positioning. As shown in Figure 4a, a circular thin PMMA disk (in green) is placed between the microfluidic chip and the upper magnet. The PMMA disk gives an even pressure distribution on the wedge surface. As shown in the cross-sectional view of the two working states of the MRE microvalve (Figure 4b), a hole is located on the PMMA disk. When the hole is positioned above the valve, the valve is disabled (open). The elastomer pushes up the wedge in the groove and the space provided by the hole above releases the pressure applied on the MRE thin layer. In this case, the valve can be used as a normal MRE micropump to drive liquid through chambers. When the PMMA is located above the wedge (no hole), the valve is enabled. The wedge is pushed down so that the applied pressure induces a deformation of the MRE, and the chamber remains sealed until the force is removed. The PMMA disk is driven by the DC motor via a gear set, which allows changing between the two valve states. We used four pieces of coloured paper and the RGB sensors to precisely locate and control the position of the hole on the PMMA disk. 

To demonstrate the ability of this pump and valve structure, a chip containing three sample inlets was fabricated. One hole was cut on the PMMA disk, which means that only one valve is released each time, to accomplish sequential pumping (Figure 4c–e and Video S3). First, the magnet rotor below is activated, meaning that the MRE valve on the right-hand side is released and works as a pump to drive the blue dye through the microchannel to the detection area (Figure 4c). Next, a push button instructs the DC motor to drive the PMMA disk, and when the RGB colour sensor detects a certain colour, the disk stops at the required position. The lower valve is then released, and the green dye is pumped out from the inlet reservoir, which can also be observed through the detection window (Figure 4d). Similar to the previous steps, the red dye is pumped through the channel to the detection area by rotating the PMMA disk to its next position (see Figure 4e). Finally, when the desired processes are finished, the platform returns to its original state and the lower rotor is switched off. A 12 V DC power supply was used for the platform, and an average current of 150 mA was measured. An average power of ~1.8 W was applied for the process, which lasted about 2 min. 

The relationship between the flow rate and the rotating speed of the rotor has also been characterised (insert plot in Figure 4e). A higher rotating speed leads to a faster traveling of the magnet and consequently induces a more effective pumping effect, reaching an average flow rate of ~120 µL/min at the speed of 150 RPM. Compared to previous studies [19], no obvious variation in flow rate was observed when the pump worked at its highest speed, which ensures the stability of the MRE-enabled microfluidic platform. With this sequential pumping chip design and flexibility of flow rates, future biological and chemical applications will be able to be performed by customising modular chip layouts in a cost-effective manner.

## 4. Conclusions

In this work, the MRE fabrication process was simplified, and three different microactuators—micropump, micromixer and microvalve—were successfully integrated into one single PMMA chip. The use of the microcontroller and sensor gives a highly controllable miniaturised platform driven by a rotating magnet rotor and a precise valve system. This enables a testing platform to be used in an insert-and-use manner. The robust combination of the controlling platform and single MRE chips has demonstrated its possibility to be utilised as a powerful tool in biological and chemical applications. Most importantly, the straightforward and low-cost fabrication process and the modularised microactuators make it useful for highly customised testing or evaluation. The remarkable abilities demonstrated by the MRE microfluidic platform can unleash the vast potential provided by modularised microfluidic systems for achieving complex lab-on-a-chip biological and chemical analysis.

## Data Availability

The data presented in this study are available in the supplementary materials. Raw data presented in this study are available on request from the corresponding author.

## Supplementary Materials

The following are available online at https://www.mdpi.com/article/10.3390/mi12060604/s1, Figure S1: Schematic of MRE microactuator production, Figure S2: Schematic of microfluidic chips integrated with MRE microactuators, Table S1: Materials’ cost estimation for a single MRE microfluidic chip, Video S1: Assembly of the controlling platform, Video S2: Demonstration for mixing and pumping, Video S3: Demonstration for sequential pumping.
