# Supplementary material for: Modular and Self-Contained Microfluidic Analytical Platforms Enabled by Magnetorheological Elastomer Microactuators
Source: Micromachines (Basel). 2021 May 23;12(6):604. doi: 10.3390/mi12060604 (PMC8224705; doi:10.3390/mi12060604)
Supplement: Supplementary file 1 [file micromachines-12-00604-s001.zip › micromachines-1218074-supplementary materials-for proof Revised-Final.pdf]

micromachines

MDPI

Supplementary Matreials

Modular and Self-Contained Microfluidic Analytical Platforms Enabled by Magnetorheological Elastomer Microactuators

Yuxin Zhang, Tim Cole, Guolin Yun, Yuxing Li, Qianbin Zhao, Hongda Lu, Jiahao Zheng, Weihua Li and Shi-Yang Tang

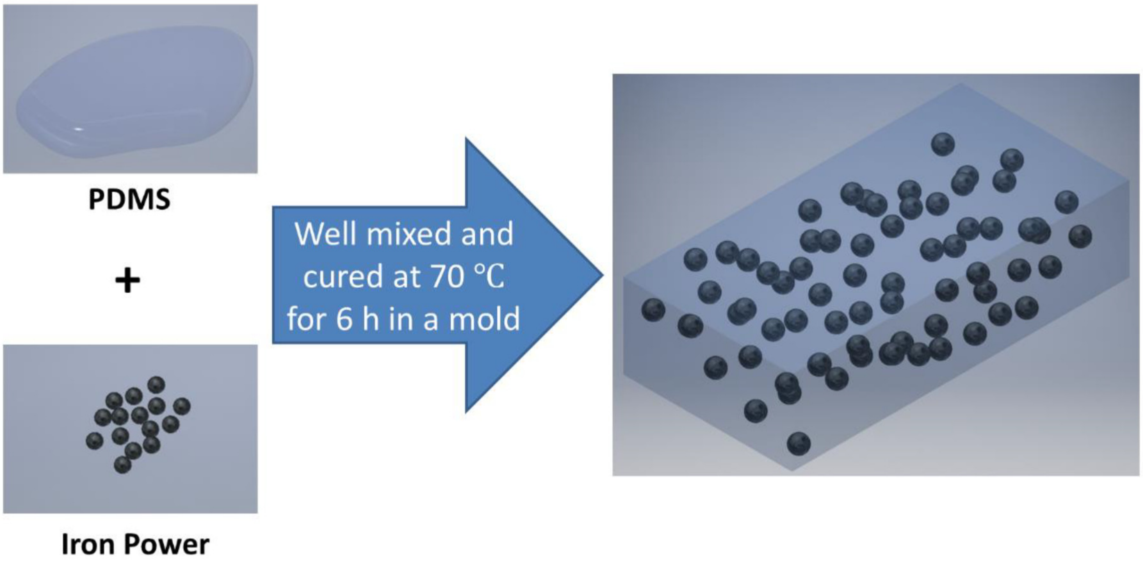

Figure S1. Schematic showing the production of MRE.

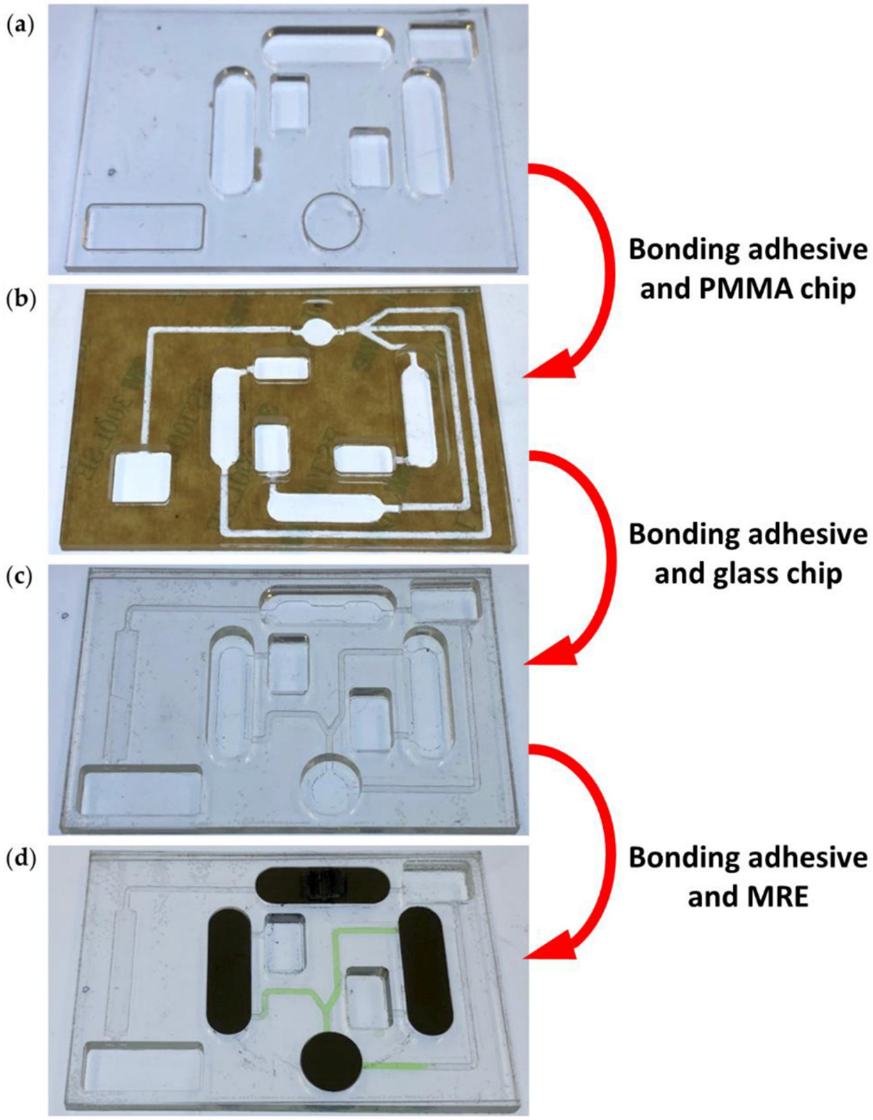

Figure S2. Illustration of microfluidics chips integrated with MRE microactuators. (**a**) Using a laser cutter to fabricate PMMA chips, (**b**) bonding double-sided adhesive and PMMA chip and then laser-cut microchannel pattern on adhesive, (**c**) bonding the chip with a glass substrate, and (**d**) plasma treating the substrate and MRE and inserting MRE microactuators into PMMA chambers.

Table S1. Materials cost estimation for a single MRE microfluidic chip.

Materials	Quantity used per chip	Cost per device (£)
Glass slide	1 unit	0.38
Double-sided adhesive	3 × 2 inches^2	0.29
PMMA chip	3 × 2 inches^2	0.80
PDMS	~2 g	~0.35
Iron microparticles	~ 8 g	~0.95
**Total**	**2.77**	
